# Introduction and comparision of three different fixation methods in the suprahepatic space in laparoscopy-assisted ventriculoperitoneal shunt for hydrocephalus

**DOI:** 10.1038/s41598-023-33566-5

**Published:** 2023-04-17

**Authors:** Qian Ding, Jinchao Wang, Haitao Fan, Wanli Jiang, Hua Guo, Hongsheng Ji, Tao Song, Shangchen Xu, Bin Liu

**Affiliations:** 1 Department of Gastroenterology, Shandong Provincial Hospital Affiliated to Shandong First Medical University, Jinan, 250021 China; 2 Department of Neurosurgery, Shandong Provincial Hospital Affiliated to Shandong First Medical University, Jinan, 250021 China; 3 First School of Clinical Medicine, Shandong University of Traditional Chinese Medicine, Jinan, 250011 China; 4 Department of Medical Imaging, Affiliated Hospital of Shandong University of Traditional Chinese Medicine, Jinan, 250011 China; 5 Department of Critical Care Medicine, Shandong Provincial Hospital Affiliated to Shandong First Medical University, Jinan, 250021 China

**Keywords:** Diseases, Medical research, Risk factors

## Abstract

Ventriculoperitoneal shunt (VPS) placement is the standard procedure in the management of hydrocephalus. The introduction of laparoscopy allows better visualization during the operation and a more reliable placement of the peritoneal terminal of the catheter, which significantly decreases postoperative obstruction and malposition rates. However, the fixation methods of the peritoneal terminal of the catheter have not been previously discussed. The indications, techniques, and complications were compared between conventional VPS and laparoscopy-guided VPS. Furthermore, same analyses were performed within the laparoscopy-guided VPS group subdivided by three different techniques of the fixation of the peritoneal terminal of catheter, including suture and ligature, titanium clip fixation, and subcutaneous fixation. A total of 137 patients with hydrocephalus who received VPS treatment was retrospectively studied, 85 of which were laparoscopy-guided, and 52 were not. The distal ends of the catheters were all placed in the suprahepatic space. At least one year (mean 28.6 months) follow-up was given postoperatively. The average duration of the whole operation was 45 min for suture and ligature, 40 min for titanium clip fixation, and 30 min for the subcutaneous fixation, respectively. Six patients (4.4%) had obstructive of the ventricular catheter in total. The success rates for the laparoscopy-assisted VPS procedure and the conventional VPS procedure were 87.1% (74/85) and 80.8% (42/52), respectively. Within subgroups of the laparoscopy-assisted VPS divided by fixation methods, the procedures were successful in 85.2% (23/27) of suture and ligation, 82.1% (23/28) of titanium clip fixation, and 93.3% (28/30) of subcutaneous fixation, respectively. Two patients had dislocated shunt tube in peritoneal end in laparoscopy group, all in the titanium clip fixation subgroups. The laparoscopy-assisted VPS insertion is an ideal shunt method for its effectiveness and lesser complication rate after operation. The subcutaneous fixation method of the peritoneal terminal of catheter might be the optimal fixation technique.

## Introduction

Ventricuoperitoneal shunt (VPS) insertion is the standard procedure in the management of hydrocephalus and yields very good results^1–4^. However, the malposition and obstruction of shunt terminal is a common postoperative complication which necessitates the replacement of the originally placed catheters. The incidence of shunt malfunction after initial placement occurs in approximately 25–35% of patients after 1 year. Up to 24% of VP-shunt patients suffer shunt-related complications and the type of dysfunction may vary depending on the age of the bypass^5,6^. Dislocation leads to reoperation after abdominal shunt in about 0.5–8% of patients^7,8^. It was shown that during the early postoperative phase, abdominal catheter misplacement or dislocation may account for up to 22% of shunt dysfunctions^6^. With the development of new minimally invasive techniques, laparoscopy-assisted VPS insertion permits direct placement of peritoneal terminal of the catheter in the suprahepatic space under direct laparoscopic visualization, which dramatically reduced the incidence of distal malfunction and revision^9,10^.

Fixation is an ideal solution for the dislocation of the distal end of the catheter. However, the outcome and techniques of the fixation have not been previously discussed. Thus, we report our experience of three fixation methods of the distal end of the catheter: suture and ligature, titannium clip, and subcutaneous fixation.

Although the new technique guaranteed the appropriate placement the peritoneal shunt terminal, the dislocation of the peritoneal terminal of the catheter and the formation of false cyst cannot be completely avoided, and the best fixation method is not determined yet. To improve the post-operative outcome and prevent peritoneal terminal catheter dislocation and omental occlusion, we compared VPS with and without laparoscopy, and compared the three ways of fixation of distal terminal of the catheter as well.

## Materials and methods

### Patient population

Between September 2004 and May 2011, 137 adult patients (72 male and 65 female, median age 40 years) underwent VPS for the treatment of hydrocephalus attributed to various causes (Table 1). Between September 2004 and May 2006, 52 VPS insertion were performed using the conventional open approach technique. Considering that laparoscopy can guarantee a better surgical visualization and decrease the distal catheter malposition rate and peritoneal organ perforation rate, thereafter we decided to introduce laparoscopy to modify our method. Eligiblility criteria include: (i) had newly diagnosed hydrocephalus necessitating VPS placement; (ii) were older than 18 years to receive laparoscopy-assisted VPS; (iii) provided written informed consent. The exclusion criteria were as follows: (i) pregnancy; (ii) inability to provide informed consent; (iii) estimated survival less than 12 months; (iv) inability to communicate. The patients receiving laparoscopy-assisted VPS were randomly assigned to three fixation methods of the distal terminal of the catheter. Of the 85 cases with laparoscopy-assisted VPS, 27 received suture and ligature fixation, 28 received titannium clip, and 30 received with subcutaneous fixation of the distal catheter, respectively. The patients’ data were retrospectively collected from their medical files, surgical reports, and follow-up notes.

**Table 1:** Demographic data and origin of hydrocephalus in 137 patients in the laparoscopy group.

Parameter	Total	Laparoscopy			Nonlaparoscopy
		Suture and ligature	Titanium clip fixation	Subcutaneous fixation	
Age (y)					
Min	9	21	18	18	9
Max	81	76	80	78	81
Median	40	49	40	43.5	36.5
Gender					
Male	72	15	15	16	26
Female	65	12	13	14	26
Disease origin (no. of cases)					
Normal-pressure hydrocephalus	49	13	10	12	14
Neoplasm	42	8	10	9	15
Post-hemorrhage	19	4	3	5	7
Post-trauma	12	1	2	3	6
Post-infection	2	0	1	0	1
Idiopathic aqueductal stenosis	13	1	2	1	9

The study was approved by the Shandong Provincial Hospital Affiliated to Shandong University Ethical Committee and the Second Affiliated Hospital of Shandong University Ethical Committee. All methods were carried out in accordance with relevant guidelines and regulations. Informed consent was obtained from all subjects and/or their legal guardian(s).

### Surgical procedure

The laparoscopy and the cranial part of the operated were performed concurrently. The proximal shunt catheter was inserted according to previously established standard operating procedure^2^. The distal terminal of VPS was placed into multi-lateral-aperture cannula and fixed with silk. One silk and a 0.5–1 cm long bow-tie are reserved for later use. A 5 mm transverse incision at inferior border of umbilicus was made into the subcutaneous tissue. The peritoneum was visualized, after puncture with a Veress needle. Through this needle, pneumoperitoneum was created and maintained at 13 mm Hg. Next, a 10-mm videoscope was introduced. Under direct visualization with the laparoscope, the distal terminal of the catheter was placed in the suprahepatic space.

**Figure 1:**
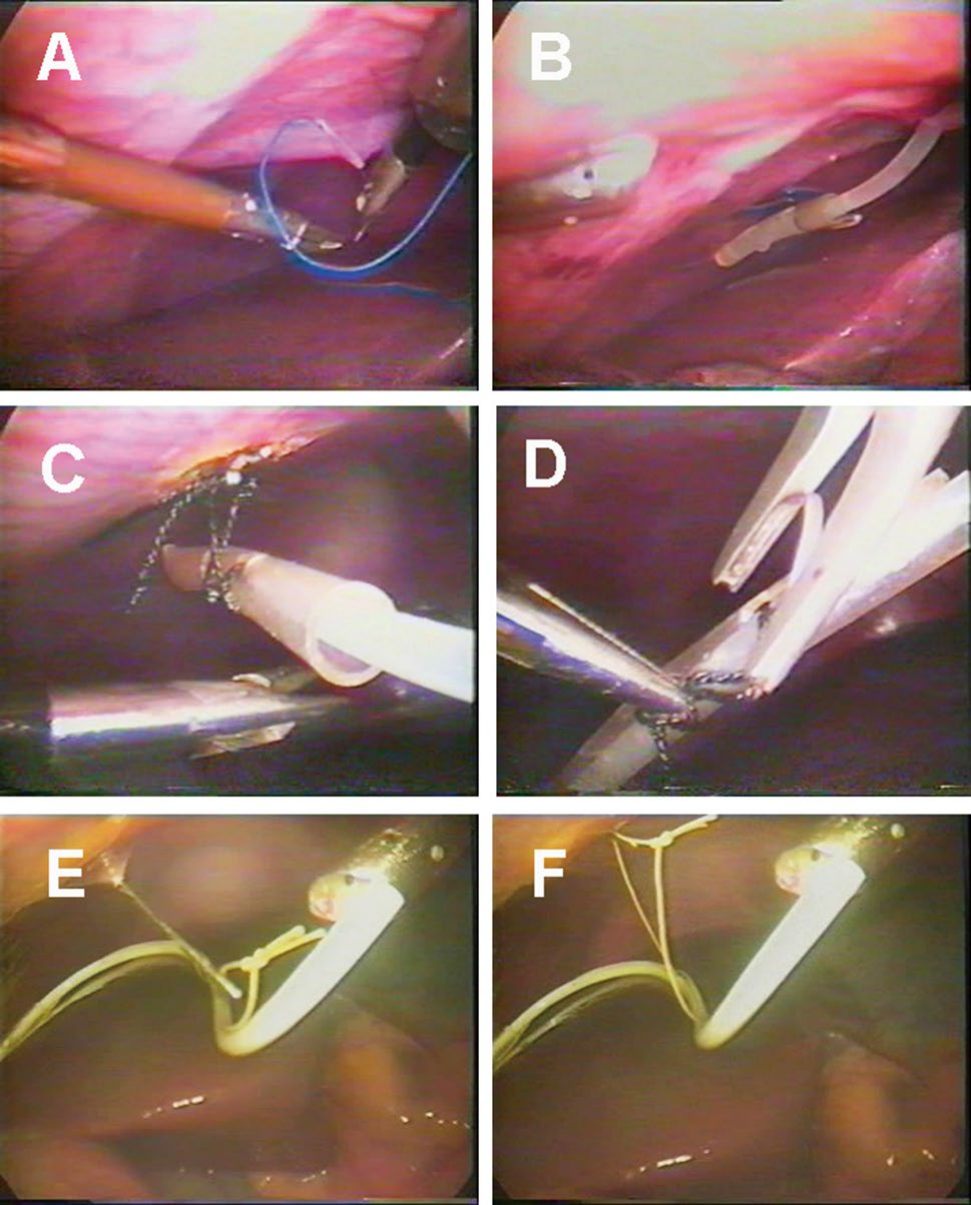
Endoscopic views showing the different fixation methods from within the peritoneal cavity. (**A**) and (**B**) Suture and ligature; (**C**) and (**D**) Titanium clip fixation; (**E**) and (**F**) Subcutaneous fixation.

With regard to fixation methods of the distal end of the catheter, there were three different surgical procedures described as follows:

Suture and ligature under laparoscope (Figs. 1A and B): three incisions were made under direct laparoscopic vision: a 5 mm subxiphoid incision (X), a 10 mm incision 5 cm inferior to xiphoid (Y), and a 5 mm subcostal incision (Z) on right anterior axillary line. Corresponding trocars were inserted through the three incisions, needle forceps were inserted through Y trocar while a curved needle with 10 cm suture silk were inserted through the Z trocar. One silk was sewed up at the abdominal surface of diaphragm at the right midclavicular 8th intercostals line, a silicone duct with a reserved ligature was introduced through X switch trocar into the suprahepatic space, then the two stitches were knotted and ligated through the silicone duct to diaphragm.

Titanium clip fixation under laparoscope (Figs. 1C and D): a 5 mm and a 10 mm trocar were inserted through X and Y incision, respectively; through Y trocar, an electro-dissecting hook was introduced to cut a 1 cm incision on the abdominal surface of diaphragm on the right 8th intercostal midclavicular line. Then through X trocar a silicone duct was inserted with a 0.5 cm bow-tie stitch, through Y trocar a titanium clip and a reserved bow-tie were inserted to fix on the dissected diaphragmatic muscle.

Subcutaneous fixation under laparoscope (Figs. 1E and F): a 5 mm or 10 mm trocar was inserted through X incision with the help of laporascope and silicone duct with a 10 cm long bow-tie was inserted, then a 0.5 cm incision was made on the right 8th intercostal midclavicular line, puncturing a 2 mm biopsy needle and holding bow-tie out of abdominal cavity, then the suture was fixed subcutaneously.

Once the distal catheter was fixed and the patency of the shunt was confirmed by visualization of CSF draining from the catheter tip (with pumping of the subgaleal valve), the laparoscope was removed and pneumoperitoneum evacuated. Finally, the trocars were removed and the incisions were closed with intracutaneous absorbable stitches.

The laparoscopic part of the operation was performed by the general surgeon at the same time that the cranial part of the procedure was performed by the neurosurgeon.

### Statistical analysis

Fisher exact test was used to analyze the difference of success rate between laparoscopy-assisted and non-laparascopy groups. One-way ANOVA followed by a Dunnett’s T3 test for multiple comparisons was used to compare the difference of success rate among subgroups divided by fixation methods in patients who received laparoscopy-assisted VPS insertion. Results were reported as being statistically significant if *P* < 0.05. Statistical analysis was performed using SPSS statistical software (Version 26.0, IBM, Armonk, NY).

### Research involving human participants and/or animals

The study was approved by the Shandong Provincial Hospital Affiliated to Shandong University Ethical Committee and the Second Affiliated Hospital of Shandong University Ethical Committee. In this study, all the operations were performed in accordance with the ethical standards and comply with the current laws of China. Informed consent was obtained from all subjects and/or their legal guardian(s). No animals were involved in this study.

## Results

A total of 137 patients (72 males and 65 females) were included in this retrospective study. The median patient age was 40 years (range: 9–81 years). The characteristics of the patients is shown in Table 1.

**Figure 2:**
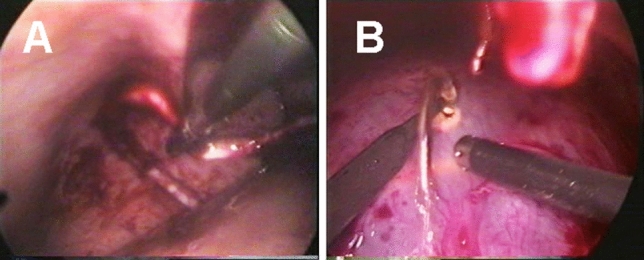
Endoscopic views showing the postoperative complications. (**A**) Obstructive shunt tube in abdominal end; (**B**) cerebrospinal fluid pseudocyst.

The laparoscopy-assisted VPS procedure was successful in 74 patients (74/85, 87.1%) and the conventional VPS procedure in 42 patients (42/52, 80.8%). The difference of success rate between the two groups were not statistically significant (*P* = 0.466). Within subgroups of the laparoscopy-assisted VPS insertion divided by fixation methods, the procedures were successful in 85.2% (23/27) of suture and ligation, 82.1% (23/28) of titanium clip fixation, and 93.3% (28/30) of subcutaneous fixation, respectively. One-way ANOVA with Dunnett’s T3 test did not show statistical significance of success rate among the subgroups divided by fixation methods (*P* > 0.05). The duration of the whole operation with suture and ligature fixation was 35–60 min, with an average time of 45 min; the titanium clip fixation was 25–60 min, with an average time of 40 min; the subcutaneous fixation was 20–50 min, with an average time of 30 min. Overall, there were 5 infection cases (5/85, 5.9%) in the peritoneal part with laparoscopy-assistance. While in the open technique VPS group, there were two cases (2/52, 3.8%) of shunt infection. There were six patients (6/137, 4.4%) who had obstruction of the shunt tube in the ventricular end (Fig. 2A) in total. Two dislocations (2/28, 7.1%) in the titanium clip subgroup led to the obstruction while none in the other two subgroups (Table 2), lower than non-laparoscopy group (4/52, 7.7%). Moreover, the only malposition and one cerebrospinal fluid pseudocyst formation (Fig. 2B) occurred in non-laparoscopy group (1/52, 1.9%). The correlation between the complication and the cause of hydrocephalus is shown in Table 3.

**Table 2:** Comparison of results between laparoscopic and nonlaparoscopic groups.

Parameter	Laparoscopy			Nonlaparoscopy
	Suture and ligature	Titanium clip fixation	Subcutaneous fixation	
No. of patients	27	28	30	52
Duration of op (min)	35–60	25–60	20–50	30–120
Complication (no. of cases)				
Infection	2	2	1	2
Dislocation of catheter in abdominal wall	0	2	0	4
Malposition of catheter	0	0	0	1
Obstruction of ventricular part	2	1	1	2
Cerebrospinal fluid pseudocyst	0	0	0	1

**Table 3:** Correlation between postoperative complications and causes of hydrocephalus.

Postoperative complication	Cause of hydrocephalus					
	Normal pressure hydrocephalus	Neoplasm	Post-hemorrhage	Post-trauma	Post-infection	Idiopathic aqueductal stenosis
Infection	2	1	1	2	1	1
Dislocation of catheter in abdominal wall	0	1	2	0	0	3
Malposition of catheter	0	0	0	1	0	0
Obstruction of ventricular part	1	3	2	1	0	0
Cerebrospinal fluid pseudocyst	0	0	0	1	0	0

## Discussion

To our knowledge, this is the first time that the fixation techniques of the peritoneal terminal of the ventriculoperitoneal shunt catheter were discussed.

The theoretical basis of VPS insertion is that the surrounding wall of abdominal cavity is covered with the peritoneum, which is composed of mesothelial cells and connective tissue with strong absorbtive capacity for cerebraospinal fluid. The first minimally invasive laparoscopic approach for percutaneous insertion of the peritoneal catheter during VPS procedures was described in 1993^11^. Thereafter, various minimally invasive techniques for the implantation of a peritoneal catheter have also been described^12–14^. In conventional open technique VPS insertion, a 5–10 cm incision was used to introduce the tube into the abdominal cavity, causing more trauma and more complications attributed to the limited vision field and the difficulty of manipulation. Laparoscopy allows minor skin scar and faster postoperative recovery, as well as the acquisition of a more direct and clearer visual field and an easier manipulation. Under the direct guidance of laparoscopy, we could guarantee that the distal terminal of the catheters is placed in the suprahepatic space, where the abdominal surface of diaphragm and the diaphragmatic surface of liver are smooth and free of omental structure, so that the terminal is difficult to be obstructed, comparing to the arbitrary placement with open technique. Moreover, with the fixation of the terminal, the dislocation and malposition of the terminal from the suprahepatic space could be avoided, preventing the re-implantation of the catheters. More importantly, damage to liver, vagina, urinary bladder, gallbladder and bowel, which have been reported in open technique^15^, could be avoided. Furthermore, the patency could be confirmed easily with the visualization provided by laparoscope^16^. Consequently, with the merits described above, the post-operative complications of VPS significantly decreased, such as the incidence rate of incision infection, incision leakage, incision herniation, ankylenteron, and bowel obstruction^12^.

The most common complication in VPS is the obstruction of the catheter. The obstruction is generally caused by the encasement by great omentum, cellulose mass blocking up, and adhesion of peritoneum. In the laparoscopy group, 2 cases (2/85, 2.4%) developed dislocation of catheters, all occurred in the titanium clip subgroups (2/28, 7.1%), which was significantly lower than non-laparoscopy group (3/52, 5.8%). Thus, we might conclude that the laparoscopy introduction could decrease the failure rate of VPS, while the titanium clip fixation stayed in question of the reliability. Another common complication is infection, leading to meningitis, ventriculitis, peritonitis, peritoneal abscess and finally the obstruction of the catheter. However, according to previous reports, the post-operative infection rate was higher in laparoscopy-assisted VPS than in open approach techniques^17,18^, varying from 2 to 10.5%. In the laparoscopy group, 5 of 85 cases (5.9%) suffered from post-operative suprahepatic abscess and among them an 81 years old female patient (1/85, 1.2%) died from sepsis with multiple organ failure half a year after the operation. The infection rate was slightly higher than in the non-laparoscopy group (2/52, 3.8%). We propose that the higher rate might be caused by insufficient skin, surgical instruments and catheter disinfections, or the host versus graft reaction. So we followed aseptic technique strictly and apply large dose antibiotics prophylacticly before and after operation, and no infection reoccurred in the last 16 cases. However, the differences were not statistically significant, which might be attributed to the relatively small sample size in the present study.

Introducing the distal part of the catheter into the peritoneal space has to be performed by opening of the peritoneum, which has to be closed afterwards to avoid any prolaps of intestinal tissue. Traditionally, fixation is on the other hand usually avoided to prevent the traction of the tube following body movements of flexion and extension. In patients without further growth such movements might be induced by stronger extensions during sportive maneuvers, elevation of both arms and extension of the ventral skin tissue. In our experience, a 25–30 cm long abdominal terminal of shunt tube is recommended, which could prevent traction or coiling of the catheter. In this study, traction of the tube was not observed during the follow-up. In case of children, it should be noted that the subcutaneous gliding is necessary to follow the principles of growth and alterations of body dimensions. Thus, fixation of parts of the catheter might induce traction and withdrawal of the peritoneal parts of the catheter, which has to be cautiously discussed in the future.

Recently, mini-laparoscopic shunt insertion is getting increasingly popular among surgeons^8,19^. This technique is also reported to be safe and bring even more benefits cosmetically compared with laparoscopic shunt placement. Schucht et al. reported that while overall shunt failure rates were similar between mini-laparoscopic shunt and laparoscopic shunt groups, the use of laparoscopic shunt placement significantly reduced the rate of distal shunt failure compared with mini-laparotomy^8^. Recently, Cherian et al. introduced single port optical access laparoscopic shunt insertion technique, which used a small cosmetic incision and obviated the need for postoperative abdominal radiological examinations^19^. However, in our experience, it is difficult to perform fixation of distal terminal of the catheters with these novel techniques, which may lead to dislocation in the long run.

Based on the present cases, we hereby concluded our experience relating to the whole operation as follows, (i) CSF should be appropriately drained after ventricle puncture in order to avoid abrupt drop of intracranial pressure inducing subdural or epidural hemorrhage^20^; (ii) The ventricular terminal of shunt tube should be inserted into the lateral ventricle no less than 11 cm so that we can ensure that the terminal lies ahead of foramen of Monro, where is free of choroids plexus, thus, it is not easy to be obstructed; (iii) During the process of the penetration of aeropetitoneum needle and trocars, they should be rotated and pushed forward simultaneously with restrained wrist strength in order to avoid damage to viscera and great vessels in abdominal cavity; (iv) The puncture position of the trocar with hooks should be carefully and appropriately selected to avoid the damage of lung or pleura; (v) Abdominal cavity part of the distal terminal should not be coiled to avoid the obstruction of the catheter; (vi) A 25–30 cm long abdominal terminal of shunt tube is recommended, otherwise it will coil in the suprahepatic space if it is too long or the traction might occur if it is too short; (vii) Since patients who received fixation of the catheter in this study were adults, fixation of the catheter should be discussed cautiously in case of children.

In our experience, we recommend the subcutaneous fixation method because the manipulation was the simplest and minimally invasive, the duration of the process was the shortest, and it is much easier to take out the catheter if necessary. But caution should be exercised to ensure that the shunt terminal is not positioned to a rather high level to avoid lung or pleura damage. Titanium fixation might not be firmly anchored so that the terminal of catheter might fall off and be encased by great omentum, with two cases (2/28, 7.1%) occurrring in this group.

Nevertheless, there are limitations in our study. First of all, the case size is relatively small. Further study with larger sample size and multiple-center cooperation is required. Secondly, the duration of follow-up was not long enough. According to the previous studies, long-term evaluation reveals that new cases of shunt infection as well as the shunt obstruction continue to occur even after years of shunt placement^3,17^. A long-term analysis is waiting in line in the future. Thirdly, since the general surgeons are not always available for the impromptu implantation of the peritoneal catheter, in the future, we hope that the neurosurgeons could master the laparoscopic technique.

## Conclusion

Laparoscopic implantation of the peritoneal terminal catheter for VPS insertion is a simple, minimally invasive, and safe technique. The fixation of the abdominal terminal of the catheter in the suprahepatic space with three fixation methods including subcutaneous fixation, suture and ligature, and titanium clip fixation were confirmed to be safe and reliable in the prevention of the dislocation and malposition of the catheter.

## Data Availability

The dataset used and/or analysed during the current study is available from the corresponding author on reasonable request.
